# Diabetes and pre-diabetes are associated with cardiovascular risk factors and carotid/femoral intima-media thickness independently of markers of insulin resistance and adiposity

**DOI:** 10.1186/1475-2840-6-32

**Published:** 2007-10-24

**Authors:** David Faeh, Julita William, Patrick Yerly, Fred Paccaud, Pascal Bovet

**Affiliations:** 1 Institute of Social and Preventive Medicine (IUMSP), University Hospital Center and University of Lausanne, Bugnon 17, 1005 Lausanne, Switzerland; 2 Department of Physiology, University of Lausanne, Switzerland; 3 Ministry of Health and Social Development, Victoria, Republic of Seychelles; 4 Division of Cardiology, Department of Medicine, University Hospital Center, Lausanne, Switzerland

## Abstract

**Background:**

Impaired glucose regulation (IGR) is associated with detrimental cardiovascular outcomes such as cardiovascular disease risk factors (CVD risk factors) or intima-media thickness (IMT). Our aim was to examine whether these associations are mediated by body mass index (BMI), waist circumference (waist) or fasting serum insulin (insulin) in a population in the African region.

**Methods:**

Major CVD risk factors (systolic blood pressure, smoking, LDL-cholesterol, HDL-cholesterol,) were measured in a random sample of adults aged 25–64 in the Seychelles (n = 1255, participation rate: 80.2%).

According to the criteria of the American Diabetes Association, IGR was divided in four ordered categories: 1) normal fasting glucose (NFG), 2) impaired fasting glucose (IFG) and normal glucose tolerance (IFG/NGT), 3) IFG and impaired glucose tolerance (IFG/IGT), and 4) diabetes mellitus (DM). Carotid and femoral IMT was assessed by ultrasound (n = 496).

**Results:**

Age-adjusted levels of the major CVD risk factors worsened gradually across IGR categories (NFG < IFG/NGT < IFG/IGT < DM), particularly HDL-cholesterol and blood pressure (p for trend < 0.001). These relationships were marginally attenuated upon further adjustment for waist, BMI or insulin (whether considered alone or combined) and most of these relationships remained significant. With regards to IMT, the association was null with IFG/NGT, weak with IFG/IGT and stronger with DM (all more markedly at femoral than carotid levels). The associations between IMT and IFG/IGT or DM (adjusted by age and major CVD risk factors) decreased only marginally upon further adjustment for BMI, waist or insulin. Further adjustment for family history of diabetes did not alter the results.

**Conclusion:**

We found graded relationships between IGR categories and both major CVD risk factors and carotid/femoral IMT. These relationships were only partly accounted for by BMI, waist and insulin. This suggests that increased CVD-risk associated with IGR is also mediated by factors other than the considered markers of adiposity and insulin resistance. The results also imply that IGR and associated major CVD risk factors should be systematically screened and appropriately managed.

## Background

Worldwide, the number of persons with diabetes mellitus (DM) is expected to double in the next 25 years and to affect more than 350 million individuals by 2030 [1]. Accordingly, there is a growing interest in identifying individuals in stages preceding overt DM in order to potentially prevent the occurrence of DM and associated complications. The majority of complications of DM are related to cardiovascular disease (CVD) and it is therefore important to assess whether pre-diabetes stages are also associated with detrimental vascular outcomes [2].

Pre-diabetes has been first described by the World Health Organization in 1980 as impaired glucose tolerance (IGT) [3]. In order to avoid the time-consuming and somewhat cumbersome measurement of 2-hour postload glucose concentrations (2hBG), the American Diabetes Association (ADA) proposed in 1997 to identify pre-diabetes as impaired fasting glucose (IFG), which relies on one fasting measurement only. In 2004, the ADA lowered the cut-off point for IFG from 6.1 to 5.6 mmol/l [4]. It has been demonstrated that both IFG and IGT are risk factors for the subsequent development of both DM [5,6] and CVD [2,5,7]. However, the respective prognostic values of IFG and IGT to predict DM and CVD risk are still controversial [5,8-11].

Because they measure different aspects of glucose metabolism, IFG and IGT have different underlying physiological and clinical significance with respect to insulin sensitivity and secretion [12-14]. Subsequently, these two categories of impairment of glucose regulation (IGR) may differently relate to CVD risk factors. However, both IFG and IGT are strongly associated with excess body weight, which in turn is associated with insulin resistance. Hence, the relationship between IGR categories and CVD risk factors or CVD outcomes may be mediated by such markers of adiposity or insulin resistance.

Intima-media thickness (IMT) of peripheral arteries (particularly carotid IMT) can be regarded as a cardiovascular risk factor since it is an independent predictor of CVD morbidity and mortality [15]. However, IMT also measures organic vascular changes -atherosclerosis- [16] and it is therefore also increasingly used as a proxy of CVD outcomes [17,18].

In this study we compared the association between IGR categories (IFG, IGT and DM) and both 1) major CVD risk factors (blood pressure, smoking, LDL-cholesterol, HDL-cholesterol) and 2) IMT (carotid and femoral IMT). We hypothesized that these associations would be substantially explained by markers of adiposity and fasting serum insulin concentration, hence that these relationships would not remain significant upon adjustment for these markers. We examined these relationships in a sample representative of the general population of the Seychelles, a rapidly developing country in health transition in the African region. The distribution of the main cardiovascular risk factors, pre-diabetes and diabetes in this population has been described previously [19-21].

## Methods

### Population

The Republic of Seychelles consists of over 100 islands located in the Indian Ocean about 2000 km east of Kenya and 2000 km north of Mauritius, in the African region. Approximately 90% of the population lives on the main island (Mahé) and most of the remaining population resides on two nearby islands. Although intermarriage has blurred racial differences in many Seychellois, it can be considered that approximately two thirds of the population is of predominantly African descent, 15% is of predominantly Caucasian, Indian or Chinese descent, and a fifth is mixed between these groups. The population can be considered as fairly urbanized in view of the high density of the population and the facts that a large proportion of the population commutes to the capital for work and three quarters of workers are employed in services [22,23]. The national gross domestic product per capita, in real terms, rose from US$ 2927 in 1980 to US$ 5239 in 2004 [24], reflecting booming tourism and fishing industries. All deaths are registered in Seychelles and vital statistics indicate a life expectancy at birth of 69 years in men and 76 years in women. In Seychelles, cardiovascular disease and AIDS currently account for 38% and 1% of all deaths, respectively [25].

### Participants

A population-based survey for cardiovascular risk factors was conducted in 2004 under the auspices of the Ministry of Health of the Republic of Seychelles. The sampling frame consisted of a sex- and age- stratified random sample of the population aged 25–64. Eligible individuals were selected from a computerized database of the entire population derived from the population census carried out in 2002, thereafter updated by civil authorities. Participants were invited to participate through a personal letter requesting them to attend a survey center on a specified day, fasting, between 7:30 and 11:00 am. Individuals were free to participate and gave written informed consent. The survey was approved by the Ministry of Health following technical and ethical reviews.

### Categories of impaired glucose regulation

Venous blood glucose (FBG) was measured with a Cholestec LDX, a point-of-care analyzer which is a reliable alternative to conventional laboratory devices [26]. The Cholestec instrument separates blood cells from plasma and measurements are therefore made on plasma. If glucose was ≥ 5.6 mmol/l, another measurement was carried out a few minutes later on capillary blood (Bayer, Ascentia Elite [27]) and the mean of both readings was used. Of note, the Ascentia Elite automatically adjusts its glucose readings to plasma values. The difference between the first FBG measurement (Cholestec) and the second FBG measurement (Ascentia Elite) was as small as -0.15 mmol/l. Individuals who were not aware of DM and with FBG ≥ 5.6 mmol/l and < 7 mmol/l were submitted to an oral glucose tolerance test (OGTT) using a meal of 75 g glucose dissolved in 300 ml water and capillary glucose (Ascentia Elite) was measured 120 min later (2hBG). Categories of impaired glucose regulation (IGR) were based on the new criteria of the ADA [4]. DM was defined as FBG ≥ 7.0 mmol/l, 2hBG ≥ 11.1 mmol/l, or a current history of antidiabetic medication. IFG refers to FBG of 5.6–6.9 mmol/l. IGT was defined as FBG < 7.0 mmol/l and 2hBG of 7.8–11.0 mmol/l. Normal glucose tolerance (NGT) was defined as 2hBG < 7.8 mmol/l. NFG refers to FBG values < 5.6 mmol/l.

### Covariates

A structured questionnaire was administered to participants by trained survey officers. Family history of DM was defined as reported DM among a first degree parent or sibling. Weight was measured with an electronic scale at 0.2 kg precision (Seca, Hamburg, Germany), height with a fixed stadiometer at 0.5 cm precision (Seca). BMI was calculated as weight divided by height squared (kg/m^2^). Waist circumference (waist) was measured at the level of the umbilicus in standing position, with individuals in light garments. Blood pressure (BP) refers to the average of the second and third of three measurements (mercury sphygmomanometer, cuff size adjusted to arm circumference). Smoking was defined as current smoking of at least 1 cigarette/day.

Serum was obtained within 2 hours of blood collection and immediately frozen to -20°C. Fasting serum insulin (insulin) was measured using commercial RIA kits (LINCO Research Inc, Missouri, USA). Blood lipids were measured using standard methods (Hitachi 917 instrument and Roche reagents). In this paper, we refer to CVD risk factors to designate major CVD risk factors, i.e. systolic blood pressure, smoking, LDL-cholesterol, and HDL-cholesterol. We used insulin as our primary marker of insulin resistance. We preferred insulin (to HOMA (homeostasis model assessment of insulin resistance) as the former is directly measured and the latter is calculated and includes a variable (fasting blood glucose) that is also used to define categories of IGR in our analyses.

### Ultrasonography

High-resolution B-mode ultrasonography was conducted in all participants above 45 years seen during a 17-week period (n = 496) as well as in a randomly selected sample (18%, n = 57) of the participants aged 35–44 years. We restricted the investigation to this age range because older persons are more likely to have atherosclerosis. Carotid intima-media thickness is a well-established surrogate marker for atherosclerotic disease that is increasingly used in observational and interventional studies [28]. All the scans and image measurements were carried out by the same investigator (P.Y.) who was blinded to the risk factor status of the participants. We used a portable ultrasound system (General Electric LogiqBook) connected with a 6–10 MHz linear array transducer. The system was equipped with a software (M'ATH, ICN-metric, Paris, France) to perform semi-automatic measures of intima-media thickness (IMT) on frame [29]. IMT was measured on the far wall of both the right and left common carotid and femoral arteries over a length of 1 cm on a reference site located 2 cm below the bifurcation. The measurements on the left and right arteries were averaged to obtain a single mean value at carotid and femoral levels and all four measurements were averaged to obtain a combined value for all four arteries. The far wall was used because of higher reproducibility and possible overestimation of the IMT of the near wall [30]. To examine the reproducibility, we made a repeat investigation in 20 randomly selected participants within few week intervals. For carotid IMT, the coefficient of variation was 4.8%, which is similar to findings in other studies [31]. For femoral IMT, the coefficient of variation was 9.2%.

### Statistical analysis

Prevalence estimates were standardized to the age distribution of the WHO [32]. We tested differences in means (± standard errors, SE) of CVD risk factors across categories of IRG with the chi-square test and the t-test, respectively. One-way analysis of variance was used to compare categorical variables between the four groups. Trends were calculated with the Stata-command "nptrend" (by Cuzick, 1985 and Altman, 1991). The associations between IFG/NGT, IFG/IGT and DM and risk factors were analyzed with multivariate linear regression. Models were adjusted for age, sex, BMI, waist, insulin, systolic BP, LDL-cholesterol, HDL-cholesterol and smoking status. We did not include triglyceride in the main multivariate analyses because: i) the independent role of triglycerides remains controversial; ii) triglyceride is generally not included as a major risk factor in CVD risk models; iii) triglyceride is strongly associated with resistance insulin and/or HDL cholesterol, as we have shown in the same data [20]. Analyses were performed with Stata 8.2 and P values < 0.05 were considered significant.

## Results

1,255 persons (568 men and 568 women) participated out of 1,565 eligible individuals, a participation rate of 80.3%. About one third (33.5%) of participants had IFG/NGT (11.6%), IFG/IGT (10.4%) or DM (11.5%). However, while the prevalence did not differ by gender for DM (11.0% in men and 12.1% in women) and IFG/IGT (11.2% in men and 9.6% in women), the prevalence of IFG/NGT was higher in men than in women (17.6% vs. 5.7%, p < 0.001). The overall age-adjusted prevalence (± SE) of family history of diabetes was 30.0 ± 1.3% (men: 26.6 ± 1.9, women: 33.2 ± 1.8). The age-adjusted prevalence of family history of diabetes tended to increase across categories of NFG (26.6 ± 1.6%, men: 22.0 ± 2.3, women: 30.4 ± 2.2), IFG/NGT (28.9 ± 3.7, men: 23.3 ± 4.6, women: 27.5 ± 6.6), IFG/IGT (36.5 ± 4.0, men: 33.1 ± 5.5, women: 40.6 ± 5.6), and DM (44.0 ± 3.7, men: 40.5 ± 5.5, women: 47.5 ± 5.0).

Table 1 shows the distribution of anthropometric, clinical and ultrasound results by categories of IGR and by sex. The results are standardized for age using 10-year age categories. Mean BMI, waist and insulin were higher in women than in men in almost all IGR categories while mean triglycerides concentration was lower in women than in men in all IGR categories (p < 0.05). In contrast, no gender difference was found in HDL cholesterol, LDL cholesterol and BP across IGR categories. Interestingly, waist tended to increase more gradually along IGR categories than BMI resulting in a larger z-value for the trend test for waist than for BMI. Also, waist tended to increase between the IFG/IGT and DM categories while BMI did not. However, the proportional increase between NGT and DM categories was almost identical for BMI and waist for both men (~13%) and women (~17%). Tests for trend were significant for all variables (we show the z-values for the tests, i.e. the log of the p value, since all p-values are < 0.01). P values for all trends tended to be stronger in women than in men.

**Table 1:** Distribution of selected clinical and metabolic factors across categories of impaired glucose regulation

Variable	NFG	IFG/NGT	IFG/IGT	DM	z-trend*
*Men*					
Clinical and metabolic data (n)	316	98	73	81	8.1
Age (years)	42.5 ± 0.6	45.1 ± 0.9	49.3 ± 1.1	52.7 ± 1.0	5.9
Body mass index (kg/m^2^)	24.6 ± 0.3	26.6 ± 0.4	26.6 ± 0.5	27.7 ± 0.6	8.2
Waist circumference (cm)	85.8 ± 0.6	92.5 ± 1.0	93.0 ± 1.4	97.5 ± 1.3	7.8
Serum fasting insulin (pmol/l)	10.8 ± 0.4	14.2 ± 0.8	19.8 ± 2.4	20.9 ± 2.1	6.9
Triglycerides (mmol/l)	0.97 ± 0.03	1.20 ± 0.07	1.56 ± 0.19	1.76 ± 0.17	2.5
LDL cholesterol (mmol/l)	3.42 ± 0.07	3.60 ± 0.13	3.83 ± 0.14	3.75 ± 0.17	-3.8
HDL cholesterol (mmol/l)	1.42 ± 0.03	1.29 ± 0.05	1.27 ± 0.06	1.20 ± 0.05	8.0
Systolic blood pressure (mmHg)	127 ± 0.9	132 ± 1.6	137 ± 1.9	145 ± 2.3	
Mean intima-media thickness (n)	104	46	48	50	2.4
Carotid (mm)	0.72 ± 0.01	0.71 ± 0.02	0.78 ± 0.03	0.75 ± 0.02	5.2
Femoral (mm)	0.93 ± 0.06	0.92 ± 0.09	1.20 ± 0.10	1.41 ± 0.13	4.9
Total (mm)	0.76 ± 0.02	0.82 ± 0.04	0.90 ± 0.03	1.04 ± 0.04	
*Women*					
Clinical and metabolic data (n)	460	47	77	103	
Age (years)	41.4 ± 0.5	52.1 ± 1.4	50.1 ± 1.0	54.4 ± 0.8	11.9
Body mass index (kg/m^2^)	27.1 ± 0.3	31.5 ± 0.9	31.8 ± 0.7	31.8 ± 0.5	8.4
Waist circumference (cm)	86.7 ± 0.6	100.1 ± 2.1	97.4 ± 1.4	101.5 ± 1.1	10.7
Serum fasting insulin (pmol/l)	13.8 ± 0.4	21.1 ± 1.9	19.4 ± 1.4	25.4 ± 2.1	8.4
Triglycerides (mmol/l)	0.82 ± 0.02	1.07 ± 0.08	1.10 ± 0.1	1.35 ± 0.08	8.8
LDL cholesterol (mmol/l)	3.44 ± 0.05	4.11 ± 0.19	3.81 ± 0.14	4.20 ± 0.14	5.8
HDL cholesterol (mmol/l)	1.40 ± 0.02	1.32 ± 0.05	1.28 ± 0.0	1.21 ± 0.04	-5.0
Systolic blood pressure (mmHg)	120 ± 0.7	137 ± 3.8	134 ± 2.1	139 ± 1.9	10.3
Mean intima-media thickness (n)	155	30	45	76	
Carotid (mm)	0.69 ± 0.01	0.75 ± 0.02	0.74 ± 0.04	0.84 ± 0.03	5.6
Femoral (mm)	0.71 ± 0.03	0.88 ± 0.09	0.84 ± 0.06	1.16 ± 0.07	6.5
Total (mm)	0.76 ± 0.02	0.82 ± 0.04	0.90 ± 0.03	1.04 ± 0.04	7.4
					
*Overall*					
Clinical and metabolic data (n)	776	145	150	184	
Age (years)	41.8 ± 0.4	47.4 ± 0.8	49.7 ± 0.7	53.7 ± 0.6	14.3
Body mass index (kg/m^2^)	26.0 ± 0.2	27.8 ± 0.4	29.0 ± 0.5	29.7 ± 0.4	9.5
Waist circumference (cm)	86.3 ± 0.4	94.3 ± 1.0	95.1 ± 1.0	99.5 ± 0.9	13.4
Serum fasting insulin (pmol/l)	12.5 ± 0.3	15.9 ± 0.8	19.6 ± 1.4	23.3 ± 1.5	11.1
Triglycerides (mmol/l)	0.89 ± 0.02	1.17 ± 0.05	1.35 ± 0.10	1.55 ± 0.09	11.2
LDL cholesterol (mmol/l)	3.43 ± 0.04	3.73 ± 0.11	3.82 ± 0.10	3.98 ± 0.11	5.8
HDL cholesterol (mmol/l)	1.41 ± 0.02	1.30 ± 0.04	1.27 ± 0.04	1.21 ± 0.03	-6.5
Systolic blood pressure (mmHg)	123 ± 0.6	134 ± 1.6	136 ± 1.4	142 ± 1.5	13.1
Mean intima-media thickness (n)	258	76	93	126	
Carotid (mm)	0.71 ± 0.01	0.72 ± 0.01	0.76 ± 0.02	0.80 ± 0.02	5.9
Femoral (mm)	0.81 ± 0.03	0.91 ± 0.07	1.04 ± 0.06	1.27 ± 0.07	8.3
Total (mm)	0.76 ± 0.02	0.82 ± 0.04	0.90 ± 0.03	1.04 ± 0.04	8.7

Values displayed are age-standardized means ± standard error.

*z-value for trend; all corresponding p-values < 0.01.

Table 2 shows the multivariate association between CVD risk factors and categories of IGR upon incremental adjustment for covariates related to adiposity (BMI and waist) and insulin resistance. All models are adjusted for sex and age, since age is strongly associated with IGR and the considered CVD risk factors (hence an important confounding factor). Incremental adjustment for BMI, waist and insulin allows disentangling confounding effects by these variables since adiposity and/or insulin resistance are known to be associated both with most of the considered CVD risk factors and IGR. Adjustment for BMI or waist reduced the magnitude of the regression coefficients between CVD risk factors and IGR only slightly. This attenuation effect tended to be larger with waist than BMI. The association between CVD risk factors and IGR categories was also slightly attenuated upon adjustment of insulin. Attenuation of the relationship between CVD risk factors and IGR was similar upon adjustment of BMI, waist or insulin. Concurrent adjustment for all three markers (BMI, waist and insulin) produced the smallest coefficients for all CVD risk factors in virtually all IGR categories. However, the attenuation of the relation between CVD risk factors and IGR categories was only marginally larger upon adjustment with all three adiposity/insulin resistance markers (BMI, waist, insulin) as compared to adjustment for any of these markers. This suggests that any of these three markers similarly reflected a common underlying mechanism.

**Table 2:** Associations between categories of impaired glucose regulation and selected cardio-metabolic risk factors

		IFG/NGT			IFG/IGT			DM		
				
	Adjustment in addition to age and sex	Coef.	SE	*p*	Coef.	SE	*p*	Coef.	SE	*p*
Triglycerides (mmol/l)										
	None	0.19	0.07	0.01	0.39	0.07	0.00	0.59	0.07	0.00
	BMI	0.14	0.07	0.05	0.33	0.07	0.00	0.52	0.07	0.00
	Waist	0.10	0.07	ns	0.31	0.07	0.00	0.46	0.07	0.00
	Insulin	0.15	0.07	0.03	0.29	0.07	0.00	0.48	0.08	0.00
	BMI, Waist, Insulin	0.09	0.07	ns	0.25	0.07	0.00	0.41	0.08	0.00
Low-density lipoprotein cholesterol (mmol/l)										
	None	0.26	0.11	0.02	0.29	0.12	0.02	0.39	0.12	0.00
	BMI	0.19	0.11	0.09	0.21	0.12	0.07	0.30	0.12	0.01
	Waist	0.15	0.11	ns	0.19	0.12	ns	0.24	0.12	0.04
	Insulin	0.22	0.11	0.05	0.20	0.12	ns	0.23	0.12	0.06
	BMI, Waist, Insulin	0.15	0.11	ns	0.15	0.12	ns	0.16	0.12	ns
High-density lipoprotein cholesterol (mmol/l)										
	None	-0.14	0.04	0.00	-0.19	0.05	0.00	-0.27	0.05	0.00
	BMI	-0.08	0.04	0.05	-0.12	0.04	0.01	-0.19	0.04	0.00
	Waist	-0.06	0.04	ns	-0.11	0.04	0.02	-0.16	0.04	0.00
	Insulin	-0.11	0.04	0.01	-0.14	0.05	0.00	-0.20	0.05	0.00
	BMI, Waist, Insulin	-0.05	0.04	ns	-0.09	0.04	0.04	-0.14	0.05	0.00
Systolic blood pressure (mmHg)										
	None	5.43	1.49	0.00	7.02	1.56	0.00	10.82	1.54	0.00
	BMI	4.15	1.48	0.01	5.55	1.56	0.00	9.14	1.54	0.00
	Waist	3.67	1.49	0.01	5.39	1.55	0.00	8.38	1.56	0.00
	Insulin	4.57	1.50	0.00	5.72	1.59	0.00	9.48	1.64	0.00
	BMI, Waist, Insulin	3.45	1.50	0.02	4.87	1.58	0.00	8.42	1.64	0.00

ns: p ≥ 0.10.

NFG: normal fasting glucose; IFG: impaired fasting glucose; NGT: normal glucose tolerance; IGT: impaired glucose tolerance; DM: diabetes mellitus; BMI: body mass index; waist: waist circumference; insulin: serum fasting insulin.

The association between CVD risk factors and IGR categories increased generally fairly monotonically over increasing IGR categories, with as much an increase in the regression coefficients between the IFG/NGT and IFG/IGT categories as between the IFG/IGT and DM categories. All the considered CVD risk factors were associated with all three IGR categories, except for CRP and IFG/NGT. However, a few associations were no longer significant upon full adjustment for adiposity and insulin resistance markers (BMI, waist, insulin). For DM, the associations remained significant for all CVD risk factors except LDL-cholesterol. For IFG/IGT, the associations remained significant for all CVD risk factors except LDL-cholesterol and CRP. For IFG/NGT, the associations remained for triglyceride and systolic BP but not for LDL-cholesterol, HDL-cholesterol and CRP. The association of IGR with BP was similar whether based on diastolic or systolic BP (results not shown).

Family history of diabetes was associated with IFG/IGT and DM, but not with IFG/NGT. Adjustment for family history of DM in addition to age, sex, BMI, waist and insulin had virtually no impact on the associations (i.e. regression coefficients) between CVD risk factors and IGR categories (results not shown). This suggests that the relationships between CVD RF and IGR are not mediated by family history. In addition, IGR categories were associated, in adjusted models, with apo B and CRP but not with apo A1 and cystatin C (results not shown).

**Figure 1:**
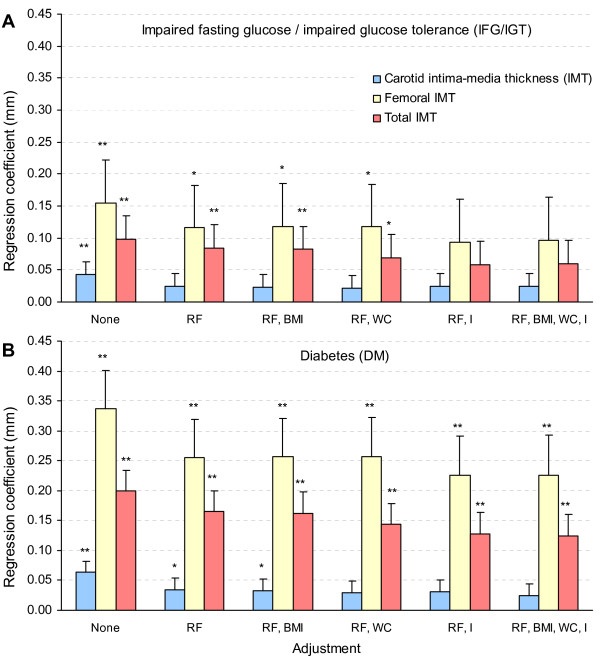
**Associations between intima-media thickness (IMT) and categories of impaired glucose metabolism upon incremental adjustment for covariates (regression coefficients with their standard errors). Panel A: impaired fasting glucose/impaired glucose tolerance (IFG/IGT); Panel B: diabetes (DM)**. * p: 0.05–0.09. ** p < 0.05. All associations between impaired fasting glucose/normal glucose tolerance (IFG/NGT) and IMT were statistically not significant and are thus not shown. None: no adjustment; RF: major risk factors (low-density lipoprotein cholesterol, high-density lipoprotein cholesterol, systolic blood pressure, smoking); BMI: body mass index; WC: waist circumference; I: serum fasting insulin.

In Figure 1, we considered carotid/femoral IMT as a marker of atherosclerosis, hence as a CVD outcome. All associations between IFG/NGT and IMT were not statistically significant and are thus not shown. The analysis examines whether IGR categories predict IMT independently of age and CVD risk factors and whether an association, if any, would be sensitive to further adjustment of markers of adiposity or insulin resistance (BMI, waist, and insulin). The data show no association with IFG/NGT, a weak association with IFG/IGT, and a stronger association with DM. All these associations were stronger at femoral than carotid levels.

Adjustment for CVD risk factors attenuated the relationship between IGR categories and IMT, which suggests that a substantial part of the effect of IGR on IMT is mediated by BP, LDL-cholesterol, HDL-cholesterol and smoking status. Further adjustment for triglyceride, in addition to the considered major CVD risk factors, left the regression coefficients virtually unchanged (results not shown). Further adjustment for BMI, waist and insulin, whether considered separately or in combination, further reduced these relationships only marginally (as assessed by the further small relative decrease in the age and CVD risk factors adjusted regression coefficients). The associations between DM and either femoral IMT or total IMT (i.e. femoral + carotid) remained significant upon full adjustment for age, sex, CVD risk factors and adiposity and insulin resistance markers. This suggests that IGR (particularly DM) is related to IMT through mechanisms other than those conveyed by these factors.

Using HOMA instead of insulin for analyses shown in Table 2 and Figure 1 resulted in slightly different regression coefficients for HOMA than insulin in some instances although results were almost identical in many instances (results not shown).

## Discussion

We found that pre-diabetes was associated with worsened levels of several major CVD risk factors as well as with increased carotid and femoral IMT independently of markers of adiposity (waist and BMI) or a marker of insulin resistance (insulin).

The smaller than expected effect of waist/BMI and insulin in explaining the relationships between IGR and the major CVD risk factors deserves several comments. First, we observed that BMI, waist or insulin attenuated the relationship between IGR categories to a fairly similar small extent and that adjusting for these factors altogether did not add substantial adjustment as compared to adjustment based on any of the three markers alone. This suggests that these three markers represent a same dimension/mechanism (e.g. insulin resistance) and that none of these markers conveys a substantial advantage in representing this dimension. In our results, waist tended however to perform slightly better than BMI. An advantage of waist over BMI has been found in some studies [33], but not all [34]. The fairly similar non-additive effect of the three considered markers (insulin, BMI, waist) has practical clinical relevance: BMI (or waist) is much simpler and less expensive to asses as compared to insulin and BMI (or waist) might be preferred to insulin for risk stratification, particularly in resource-constrained settings.

Several factors may underlie the smaller than expected effect of the considered markers (BMI, waist, insulin) on the relationship between IGR and major CVD risk factors. First, this may reflect the known fact that only a sub-group of obese persons are insulin resistant and are at risk for developing IGR [35] and that obese individuals without insulin resistance have only marginally increased CVD risk [35,36]. Second, BMI, waist and insulin may only imperfectly represent insulin resistance and the effect of such markers on the relationship between IGR and major CVD risk factors might have been larger, had we used better indicators of insulin resistance. However, better markers of insulin resistance, such as the euglycemic clamp, would require complex measurements that are not practical for epidemiological studies or usual clinical practice. Third, insulin resistance is a broad description underlying many different altered physiological factors. In particular, insulin resistance has been associated with a variety of pathophysiological effects related to abdominal fat, including cytokines secretion and inflammatory cell migration [37]. For instance, the proinflammatory cytokine TNF-alpha may impair intracellular insulin signaling independently of insulin [37]. BMI, waist and insulin may therefore only poorly correlate with such finer physiological factors. In particular, insulin may not be a reliable marker for insulin resistance and subsequent atherosclerosis [38]. This could particularly be the case in diabetic persons with depleted insulin secretion (whether type 1 or type 2 DM). However, a moderate effect of adiposity/insulin in explaining the relationship between IGR and major CVD risk factors is at odds with trials showing decreased incidence of DM among pre-diabetic persons who decreased their weight through lifestyle interventions [39] or through bariatric surgery [40]. A possible explanation underlying this contradiction is that these interventions not only decreased fat mass or insulin but also improved other factors which may directly improve insulin resistance, e.g. physical activity, nutritional patterns or secretion/action of several hormones (e.g. incretins).

Similarly to the association with major CVD risk factors, the relationship between IGR and IMT was only partially explained by waist, BMI and insulin. In other studies, carotid IMT was associated with 2hBG but with neither fasting glycemia nor a insulin sensitivity index [41,42]. Consistent with our results, IMT remained significantly associated with 2hBG upon adjustment for major CVD risk factors, waist, BMI and insulin sensitivity index [41]. Interestingly, we found that the relationships between IGR and IMT tended to be larger at femoral than carotid levels independently of adjustment. We are not aware of any other study that has investigated these associations between IGR and IMT at femoral level. However it was recently shown that metabolic syndrome components impacted selectively on IMT at the femoral site: insulin and triglyceride concentrations were strongly associated with femoral IMT but not with carotid IMT [18]. These findings suggest the usefulness of femoral IMT for assessing CVD outcomes [18,43].

In addition, the relationships between IGR and either major CVD risk factors or IMT may be linked to several specific mechanisms. It is shown that atherosclerosis is accelerated by insulin resistance and DM [38]. Atherosclerosis, which also encompasses an inflammatory process [44], is in turn related to several factors associated with adiposity and/or insulin resistance. These factors include numerous adipokines [45] that are released or modulated by adipose tissue. From another perspective, insulin resistance has also been shown to alter the endothelial function, which can result in impaired production of NO [46], reduced blood flow, pro-inflammatory state and pro-thrombotic state [46,47]. Furthermore, hyperglycemia can also induce atherosclerosis independently of insulin, e.g. through glycation of proteins and lipids and by increasing oxidative stress [44].

We found that IGT was associated with major CVD risk factors or IMT more strongly than IFG. This different significance of IFG and IGT is consistent with other studies showing a stronger association of IGT than IFG with CVD risk factors [48] and with IMT [41,49], but not with a recent prospective study linking CVD and total mortality at least as strongly with IFG as with IGT [7]. A greater impact of IGT than IFG may relate to the facts that subjects with IFG and IGT are more likely to be insulin resistant whereas subjects with IFG and NGT are more likely to have insufficient insulin secretion [48,50,51]. It has been suggested that a pro-inflammatory state leading to CVD seems restricted to those individuals with IGR who are insulin resistant, measured by an insulin sensitivity index [52] or HOMA-IR [53].

Several limitations of this study need do be considered. First, we did not perform OGTT in individuals with FBG < 5.6 mmol/l and we could have missed a few cases of DM and IGT in persons with NFG but pathologically high 2hBG. The number of such cases is however expected to be small [54]. A second limitation is related to the cross-sectional nature of our study which precludes defining causal relationships. Third, we may not have captured insulin resistance optimally with BMI, waist and insulin. insulin was shown to relate only moderately with insulin resistance measured by euglycemic clamp [55]. Also, BMI and waist are only proxy measures of total adiposity and intra-abdominal adiposity. Yet, this study adds to the limited information on the associations between pre diabetes, on the one hand, and CVD risk factors and peripheral artery IMT, on the other hand. We are not aware of any previous study that has examined this issue in a population in the African region.

## Conclusion

Our data show that IGT and to a lesser extent IFG (in addition to DM) are associated with impaired cardiovascular conditions (whether risk factors or IMT) and that these associations are only moderately mediated by markers of adiposity and insulin resistance. These findings provide further evidence for increased cardiovascular risk associated with pre-diabetes (the increased cardiovascular risk associated with DM is well established) and further stress the need for early screening and management of pre-diabetes.

## Abbreviations

BMI: body mass index; BP: blood pressure; CRP: C-reactive protein. CVD: cardiovascular disease; DM: diabetes mellitus; FBG: fasting blood glucose; HDL-C: high-density lipoprotein cholesterol; HOMA-IR: homeostasis model assessment of insulin resistance; IFG: impaired fasting glucose; IGT: impaired glucose tolerance; IMT: intima-media thickness; LDL-C: low density lipoprotein cholesterol; OGTT: oral glucose tolerance test; 2hBG: 2-hour postload blood glucose

## Competing interests

The author(s) declare that they have no competing interests.

## Authors' contributions

DF led the analysis of the data and the write up of the manuscript. JW coordinated several aspects of the survey and reviewed the manuscript. PY conducted ultrasonography on all participants and reviewed the manuscript. FP assisted in the interpretation of data and reviewed the manuscript. PB led the organization of the survey, assisted with the analysis and interpretation of the data and with the write up of the manuscript. All authors read and approved the final manuscript.
